# A County Tuberculosis Scheme

**Published:** 1921-08-13

**Authors:** 


### A County Tuberculosis Scheme.

### CO-OPERATION OF GENERAL PRACTITIONERS.

The Health Committee of the Essex County Council has prepared a new tuberculosis scheme by which it is hoped to secure a closer co-operation between the general practitioners and the tuberculosis officers, and so secure the notification of the disease or its suspected existence at an earlier stage when it is more readily curable, and to generally increase the effectiveness of the existing arrangements. Local medical and panel committees have undertaken to act in full co-operation with the chief tuberculosis officer in carrying out the proposals and doing their utmost to prevail upon other members of the profession to do likewise. The scheme is for the tuberculosis officers to act as consultants. The old procedure for ordinary symptomatic treatment by the tuberculosis officers to patients will be abandoned, but the officers will give guidance and special treatment to those who cannot pay a general practitioner or will not go to a doctor. Patients suffering from tuberculosis who are referred to their own doctor will be seen by the tuberculosis officer as often as is deemed necessary. The tuberculosis nurses and health visitors will visit the homes, and remedial measures to be carried out by them will be under the direction of the general practitioner concerned. A general practitioner will be at liberty to seek a consultation with any tuberculosis officer in any case, or of suspected disease. School medical inspectors should, on finding a child suffering from the disease, advise the parent to consult the family doctor, at the same time reporting such cases. Dispensaries are to be regarded as places for consultation, diagnoses, and, after consultation with the general practitioners, the giving of special forms of treatment. Observation beds are to be provided in connection with dispensaries.

